# Kinetics of SARS-CoV-2 Viral Load in Hospitalized Patients

**DOI:** 10.3390/pathogens13050429

**Published:** 2024-05-20

**Authors:** Alessandra Panico, Francesco Bagordo, Emanuela Nolasco, Tiziana Grassi, Annagrazia Bianco, Floriano Indino, Federica Taurino, Antonella De Donno, Giambattista Lobreglio

**Affiliations:** 1 Department of Experimental Medicine, University of Salento, 73100 Lecce, Italy; alessandra.panico@unisalento.it (A.P.); antonella.dedonno@unisalento.it (A.D.D.); 2 Department of Pharmacy-Pharmaceutical Sciences, University of Bari Aldo Moro, 70121 Bari, Italy; francesco.bagordo@uniba.it; 3 Clinical Pathology and Microbiology Unit, Vito Fazzi General Hospital, 73100 Lecce, Italy; emanuela.nolasco@asl.lecce.it (E.N.); annagrazia_bianco@libero.it (A.B.); floriano.indino@gmail.com (F.I.); federica.taurino@asl.lecce.it (F.T.); patologiaclinica.polecce@asl.lecce.it (G.L.)

**Keywords:** COVID-19, cycle threshold, SARS-CoV-2, viral load

## Abstract

The rapid and accurate detection of infectious people is crucial in controlling outbreaks. The aim of this study was to evaluate the kinetics of the viral load expressed as Ct in COVID-19 hospitalized patients. Nasopharyngeal swab specimens were collected for RT-PCR testing. Forty-one subjects were recruited, of which 48.8% developed severe symptoms and 51.2% showed milder symptoms. The distribution of Ct values measured from the symptom onset showed that the kinetics of the viral load decreased with increasing time. A Ct of 25 (high viral load) was reached after a mean of 9.9 ± 4.8 days from the symptom onset, without a significant difference between patients with severe (10.9 ± 5.7 days) and milder (9.0 ± 3.9 days) symptoms. In 65.8% of cases, a high viral load was maintained for more than 7 days from the symptom onset, especially in patients with severe symptoms (70.6%). A Ct of 30 (moderate viral load) and of 38 (low viral load) were reached after a mean of 16.1 ± 8.1 and 28.5 ± 22.4 days from the symptom onset, respectively, with a significant difference between patients with severe (Ct = 30:17.9 ± 9.8 days; Ct = 38:34.6 ± 29.6 days) and milder (Ct = 30:14.3 ± 5.8 days; Ct = 38:22.7 ± 9.9 days) symptoms. These results provide an understanding of the viral kinetics of SARS-CoV-2 and have implications for pandemic control strategies and practices.

## 1. Introduction

Coronavirus disease 2019 (COVID-19) represents a significant public health emergency. In early 2020, severe acute respiratory syndrome coronavirus 2 (SARS-CoV-2) spread rapidly across many countries, causing a new pandemic [1]. Different forms of the disease can occur, with more or less severe symptoms, including pneumonia [2].

Several measures have been put in place to contain the spread of the virus, including social distancing, the use of masks, the isolation of cases, and the limitation of social and economic activities; similarly, to limit the severity of the disease, effective therapies and vaccines have been developed, contributing significantly to the reduction in the number of deaths and infections [3,4,5].

In order to implement effective public health measures, it is essential to gain a deeper understanding of the duration of infectivity. This information will also impact infection control practices for the early containment of emerging outbreaks.

To date, the diagnosis of COVID-19 is mainly based on the detection of SARS-CoV-2 through RT-PCR assay on nasopharyngeal swab specimens [6,7]. The viral load is quantified by the cycle threshold (Ct) values, which are inversely proportional to the amount of RNA virus copies and have been used as an indicator of the viral load [8].

Many PCR assays use 40 as a cut-off for the Ct value to consider the test positive, allowing the detection of even very few starting RNA molecules [9].

A recent study analyzed the Ct values of positive samples reported during the first and second waves of the pandemic and categorized high, moderate, and low viral load Ct values as below 25, between 25–30, and over 30, respectively. They suggested that a greater proportion of positive samples with a low Ct value (high viral load) might serve as an early indicator of an imminent surge [10].

Several studies observed a correlation between the low Ct value measured in respiratory samples and the infectivity of SARS-CoV-2, which is related to the transmissibility and, consequently, the rapid surge of cases [11,12]. Moreover, the ability of the virus to replicate can be assessed in cultured cells [11,13]. A recent study evaluated the correlation of the Ct value with the growth of SARS-CoV-2 in a cell culture. The data indicated that the virus infectivity was reduced when the Ct value was >24 and that for each unit increase in the Ct value, the odds ratio for recovering the virus in the cell culture decreased by 32% [13]. Nevertheless, other studies showed that the virus replicated, and so it was viable, even in samples with higher Ct values. For example, Singanayagam et al. [14] observed a strong relationship between the Ct value and the ability to recover an infectious virus. They found that 8.3% of the samples with a Ct value > 35 yielded SARS-CoV-2 in a viral culture. La Scola et al. [11] observed a stepwise decrease in the culture positivity rate with increasing Ct values. Specifically, 12% of samples with a Ct of 33 showed growth in a viral culture. However, no virus was recovered in samples with a Ct ≥ 34.

Several studies reported that the highest values of viral loads are typically detected during the first week of symptoms [15,16,17] followed by a subsequent continuous decrease. Generally, the patients exhibited low Ct levels during the infection period, which were lower during the first week than the second one [15].

The aim of this study was to evaluate the kinetics of the viral load expressed as Ct in hospitalized patients affected by COVID-19 or who contracted it during a hospital stay for other causes.

## 2. Materials and Methods

### 2.1. Study Design and Participants

This study was conducted as part of the “COVID-19 Research Project”, a collaborative initiative between the Local Health Authority (ASL) of Lecce and the University of Salento. During the year 2022, a group of hospitalized patients from different wards of the “Vito Fazzi” Hospital in Lecce (Italy) were tested for the qualitative detection of the SARS-CoV-2 RNA by RT-PCR.

Each patient was subjected to molecular testing upon entry to the hospital (1–16 days after the symptom onset) and at different time points (up to 32 days) depending on the course of the disease.

The onset of symptoms and the relative post symptoms time were determined through an objective evaluation by the doctor carried out on the basis of the anamnestic analysis and the patient-reported information (the onset of fever or respiratory symptoms) at the time of hospitalization.

For each subject, the age, sex, type of inpatient ward, any COVID-19 symptoms, and dates of testing were recorded. A signed informed consent form was obtained from all the participants for research data collection.

### 2.2. Definitions

COVID-19 patients were individuals who tested positive for SARS-CoV-2 via a nasopharyngeal swab test result, as determined by laboratory-based reverse transcriptase real-time PCR. The term “pauci-symptomatic” was used to describe those who exhibited symptoms such as fever, cough, sore throat, and fatigue. Patients with “mild symptoms” were defined as those who presented with fever, respiratory symptoms, and mild pneumonia. Finally, patients with “severe symptoms” were identified as those who exhibited difficulty breathing, hypoxia, abnormal blood gas analysis, and severe pneumonia [18].

### 2.3. Molecular Assay

Nasopharyngeal swab specimens were collected from each patient for the molecular test. The detection of SARS-CoV-2 RNA was performed using the Cobas^®^ SARS-CoV-2 assay on the 6800 platform (Roche Diagnostics, Indianapolis, IN, USA). The nucleic acid was initially extracted from the samples and purified automatically. Subsequently, it was amplified by PCR and detected.

The target nucleic acid was selectively amplified using target-specific forward and reverse primers for the ORF1 a/b non-structural region. Furthermore, a conserved region within the structural protein envelope E-gene was selected for the purpose of detecting SARS-CoV-2. Testing was performed according to the manufacturer’s instructions, and the cut-off to discriminate negative tests was set at a Ct > 38.

### 2.4. Statistical Analysis

The data on the subjects’ characteristics and the results of the molecular analysis were entered into a Microsoft Excel database and statistically analyzed using MedCalc Software version 12.3 (MedCalc Software bvba, Ostend, Belgium).

The features of the study population were statistically described as means, standard deviations, or frequencies (%). A scatter graph was used to show the distribution of Ct values measured in mild/pauci-symptomatic patients and in patients with severe symptoms against time (days) from the symptom onset.

The Ct values were used to construct a linear regression model for each patient to estimate the Ct value as a function of time since symptom onset. Specifically, the times (days) required to reach Ct values of 25, 30, and 38 were calculated for each patient according to the relative linear equation constructed on the basis of the results of the molecular test. Depending on the course of the disease, the number of molecular tests performed on each patient varied from two to ten. The estimate was calculated on the overall sample and in the subgroups of mild/pauci-symptomatic patients and patients with severe symptoms. Since the distribution of values was not normal, differences between the groups were assessed using the nonparametric Mann–Whitney test. A box plot was used to show the distribution of the estimated time (days) to reach a Ct of 25, 30, and 38. Outliers were determined using Tukey’s fences (K = 1.5).

### 2.5. Ethical Aspects

This study received a favorable decision from the Ethical Committee of the Lecce Local Health Authority (ASL/LE) (Resolution 29 May 2020, No. 557). Patient information was collected anonymously and reported as aggregate data in accordance with Italian laws (Legislative Decree 30 June 2003, No. 196).

## 3. Results

A total of 41 subjects were included in the study: 22 (53.7%) were males and 19 (46.3%) were females. The average age was 74.0 ± 13.8 years (range 40–96). As for symptoms, 20 (48.8%) patients developed severe clinical features, with pneumonia and breathing failure, while 21 (51.2%) subjects showed mild symptoms or were pauci-symptomatic. There were 33 patients hospitalized because of COVID-19 (80.5%), while there were 8 patients hospitalized for other reasons (i.e., orthopedic trauma, cardiovascular diseases) (19.5%). The patients with severe symptoms were mostly males (15, 75.0%), the average age was 77.3 ± 13.9 years, and almost all of them were hospitalized because of COVID-19 (19, 95.0%). The patients with mild or less symptoms were mainly females (14, 66.7%) who were hospitalized for COVID-19 (14, 66.7%) and had a mean age of 70.7 ± 13.8 years (Table 1).

**Figure 1:**
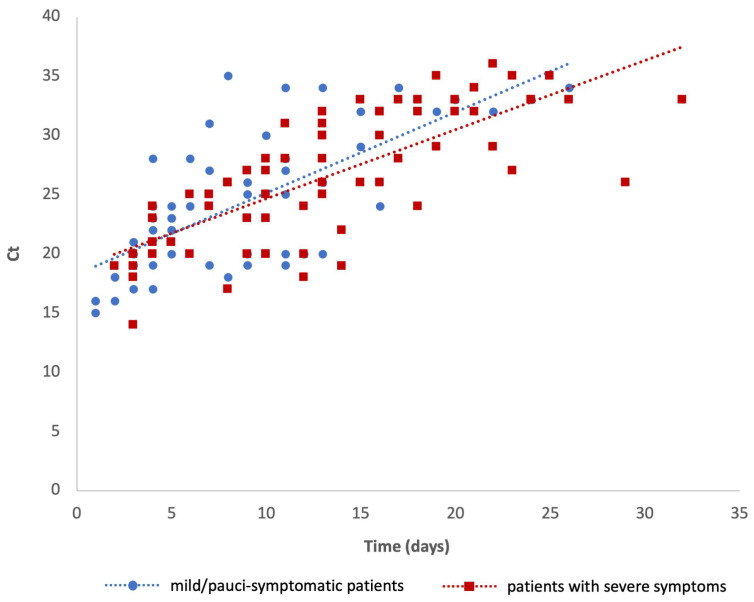
Scatter graph showing the viral load expressed as Ct values in mild/pauci-symptomatic patients and in patients with severe symptoms against time.

The scatter graph shows the distribution of the Ct values measured in both groups of patients and describes the viral load kinetics at each testing from the symptom onset (Figure 1). The regression equations (mild/pauci-symptomatic patients: y = 0.6848x + 18.255, R^2^ = 0.5041; patients with severe symptoms: y = 0.5819x + 18.775, R^2^ = 0.5603) show that the Ct values tend to increase with increasing time after symptom onset more slowly in patients with severe symptoms than in mild/pauci-symptomatic patients.

**Figure 2:**
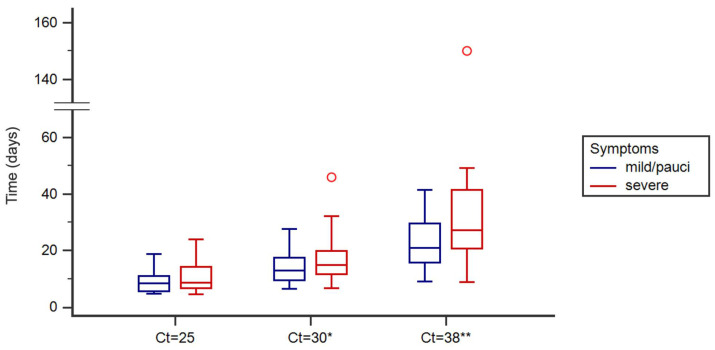
Box plot showing the distribution of values related to estimated time (days) needed to reach a Ct of 25, 30, and 38 in mild/pauci-symptomatic patients (N = 21 patients) and patients with severe symptoms (N = 20 patients). Outliers, shown as red circles, were determined using Tukey’s fences (K = 1.5). Significant differences (*p* < 0.05) in the distribution of values for Ct = 30 (*) and Ct = 38 (**) between the two groups of patients.

The box plots show the distribution of the values related to the estimated time (days) needed to reach a Ct of 25, 30, and 38 in mild/pauci-symptomatic patients and in patients with severe symptoms and describe the viral load trend from the symptom onset (Figure 2).

For the patients who had a high viral load (Ct ≤ 25) in the first phase of the disease (n = 38), a Ct of 25 was reached after a mean of 9.9 ± 4.8 days from the symptom onset, without a significant difference between the patients with severe symptoms (10.9 ± 5.7 days) and mild/pauci-symptomatic patients (9.0 ± 3.9 days). These patients maintained a high viral load (Ct ≤ 25) for more than 7 days from the onset of symptoms in 65.8% of cases (61.9% in mild/pauci-symptomatic patients and 70.6% in patients with severe symptoms). The estimated maximum value of days with Ct ≤ 25 was 18.9 days for mild/pauci-symptomatic patients and 24.1 among patients with severe symptoms.

For all the patients, a Ct of 30 was reached after a mean of 16.1 ± 8.1 days and a Ct of 38 was reached after a mean of 28.5 ± 22.4 days from the symptom onset. In particular, the patients with severe symptoms took a longer time in days (*p* < 0.05) (17.9 ± 9.8 days, max 46.0 days) to reach a Ct = 30 and a Ct = 38 (34.6 ± 29.6 days, max 150.0 days) than the mild/pauci-symptomatic patients, who took 14.3 ± 5.8 days (max 27.6 days) and 22.7 ± 9.9 days (max 41.5 days), respectively. In addition, the time to negativization (Ct > 38) was found to not be associated (*p* > 0.05) with the patient’s sex and age.

Mild/pauci-symptomatic patients exhibited a high number (N = 5) of negative swab tests starting from the second week, with the highest number (N = 6) observed in the third week from the symptom onset. In contrast, the patients with severe symptoms exhibited a low number (N = 2) of negative swab tests in the second week and the highest number (N = 5) in the fourth week.

## 4. Discussion

An effective public health control measure for the early containment of an epidemic caused by directly transmitted infections involves the early identification and isolation of infected persons as well as the tracing, testing, and quarantine of their contacts [19]. To prevent the spread of COVID-19, it is important to identify and isolate contagious people. In particular, it is crucial to ensure that individuals with a high viral load are isolated and unable to transmit the virus to others.

A critical parameter in the implementation of effective control measures is the duration of infectiousness, which is determined by viral load kinetics and the duration of viral shedding [20].

The use of RT-PCR and Ct values was identified as a means of obtaining a more complete picture of the viral dynamics exhibited by patients diagnosed with COVID-19. A higher Ct value is associated with a low viral RNA load and a reduced risk of infection transmission [21]. Ct values represent a basic and readily accessible instrument for forecasting and modeling epidemiological dynamics at the population level [22].

Many current hygiene concepts are based on the statistical assumption that the probability of infectivity decreases proportionally as the Ct value increases. While this is true under certain circumstances, the conclusion that patients with elevated Ct values are no longer capable of transmitting infectious viral particles has the potential to negatively impact the effective management of the pandemic until the minimal infectious dose is reliably determined [23].

Patients infected with SARS-CoV-2 are likely to be most contagious in the first week of illness, underlining the importance of prompt isolation with the early onset of symptoms. A number of studies indicated that viral load peaks occur during the pre-symptomatic phase of the disease or at the onset of symptoms [16,17,24,25,26]. This evidence provides a rationale for the efficient spread of SARS-CoV-2. In the present study, data collected from a group of patients hospitalized for COVID-19 or who contracted it during their hospital stay, showed the kinetics of the viral load, which decreased with increasing time, suggesting a high level of infectivity, especially in the initial stages of the disease. This finding is supported by the results from contact tracing studies, which revealed that the highest risk of transmission occurs in the earliest stages of the disease, namely a few days before and after the symptom onset [27,28]. In particular, in the patients with an early high viral load (Ct ≤ 25), we observed a mean of 9.9 ± 4.8 days from the symptom onset to reach a Ct = 25. In this group of patients, 65.8% of cases maintained a high viral load (Ct ≤ 25) for more than 7 days from the onset of symptoms, especially those with severe symptoms (70.6%), who showed a maximum of 24.1 days. 

Overall, a Ct of 30 was reached after a mean of 16.1 ± 8.1 days and a Ct of 38 was reached after a mean of 28.5 ± 22.4 days from the symptom onset.

A recent study sought to determine whether a Ct value cut-off of 30 reflected the infectivity potential among close contacts of COVID-19-positive cases. The authors found that more than half of the positive cases followed up had at least one secondary case, with a median positivity rate of 12.5%. A significant correlation was observed between the Ct value cut-off of 30 and secondary transmission. Those with a Ct value <30 exhibited a 1.5-fold increased risk of secondary transmission. A significant difference was highlighted in the median positivity rate between the close contacts of positive subjects with a Ct value <30 and those with a value >30 [19].

In more severe cases, viral RNA is typically detected for a longer time. Several studies demonstrated that viral RNA can be detected in respiratory samples as early as one to three days before the symptom onset. In mild cases, the viral load tends to increase up to seven to ten days after the symptom onset, while in more severe cases, the viral load reaches a peak approximately eleven days after onset, followed by a gradual decline over time [25,29]. In the present study, no differences were found between patients with severe symptoms and mild/pauci-symptomatic patients regarding the length of time during which the viral load was high (Ct < 25) but were only observed in the periods when the viral load began to decrease (Ct = 30 and =38).

Several studies observed that viral shedding persists for more than 20 days following the onset of symptoms, with some cases extending up to 63 days, and it appears to continue beyond the resolution of symptoms [26,30,31,32].

Xiao et al. [33] reported that 56 hospitalized patients affected by mild to moderate COVID-19 disease can remain positive for up to 5 weeks following the symptom onset. The median interval between the symptom onset and the occurrence of a negative test result was 24 days. Some factors, such as older age and comorbidities (i.e., diabetes and hypertension) were associated with prolonged positive test results. Overall, our data showed a period of 28.5 ± 22.4 days from the symptom onset to reach negativity (Ct = 38); in particular, this time was elongated for patients with severe symptoms with a mean of 34.6 ± 29.6 days (up to a maximum of 150.0 days). The distribution of patients according to time to negativization (Ct > 38) also shows that in general, mild/pauci-symptomatic patients reach negativization before patients with severe symptoms. Among the latter group, there are cases of patients with high infectivity even after week 6, while for mild/pauci-symptomatic patients negativization occurs for all by week 6.

The emerging evidence indicates a correlation between virus persistence and disease severity and outcome [30,34,35]. This is in line with the viral load dynamics observed in other viruses, including influenza, MERS-CoV, and SARS-CoV, whereby severe forms of the disease were also associated with prolonged viral shedding [36,37]. Our data also showed that patients with severe symptoms needed a higher number of days to reach a moderate viral load (Ct > 30) than mild/pauci-symptomatic patients, suggesting a faster viral clearance for the latter ones. However, more studies are needed in order to understand the duration of viable virus in patients with severe disease.

This study has some limitations. First, the sample size of this study was small and included only hospitalized patients; therefore, larger studies are needed to fully establish the kinetics of the viral load. In particular, the patients were tested after the onset of symptoms; therefore, we were not able to determine the peak of the viral load. Second, we did not isolate the virus in culture from the patient samples. Although RT-PCR is the most commonly employed method for detecting SARS-CoV-2 genome sequences and to discriminate between positive and negative subjects, viral culture represents one of the best indicators of infectivity [38]. The propagation of the virus from clinical samples serves to confirm the presence of an infectious virus. However, this method is not widely available, requires biosafety level 3 facilities, and the results are not available in a timely manner. Third, it is challenging to determine a Ct value threshold that can accurately predict infectivity, as the viral load present in clinical samples can vary due to the emergence of new SARS-CoV-2 variants [39]. Finally, it is widely acknowledged that Ct values cannot be directly compared across different testing platforms due to variability in the target design, the nucleic acid extraction method and efficiency, and the amplification technique [40].

## 5. Conclusions

The rapid and accurate detection of SARS-CoV-2 is of paramount importance in the control of the COVID-19 outbreak in the community. The use of Ct values could provide an effective indicator for the detection of contagiousness and may prove useful in informing decision-making on infection control. The results reported in the present study provide an understanding of the viral kinetics of SARS-CoV-2 and have implications for pandemic control strategies and practices. We reported for all patients the mean time to reach a Ct of 25, 30, and 38, set as high, moderate, and low viral load. In particular, the patients with severe symptoms showed a prolonged period in which the viral load remained elevated and needed a higher number of days to reach negativity than the patients with milder symptoms. 

It is possible that the indications provided by the Ct values may be associated with several benefits, including insights into the prognosis and consequences of the infection. Nevertheless, further investigations combining RT-PCR and culture studies are warranted to elucidate the relationship between SARS-CoV-2 detection, the Ct value, and transmission and to identify reliable predictors of the infectious status of COVID-19 patients.

## Figures and Tables

**Table 1:** Descriptive characteristics of patients with severe symptoms and mild/pauci-symptomatic patients.

Variables	Patients with Severe Symptoms (n = 20)	Mild/Pauci-Symptomatic Patients (n = 21)
Male	15 (75.0%)	7 (33.3%)
Female	5 (25%)	14 (66.7%)
Mean age (years)	77.3 ± 13.9	70.7 ± 13.8
Hospitalized for COVID-19	19 (95.0%)	14 (66.7%)
Hospitalized for other reasons	1 (5.0%)	7 (23.8%)

## Data Availability

The raw data supporting the conclusions of this article will be made available by the authors upon request.

## References

[1] Muralidar S., Ambi S.V., Sekaran S., Krishnan U.M. (2020). The emergence of COVID-19 as a global pandemic: Understanding the epidemiology, immune response and potential therapeutic targets of SARS-CoV-2. Biochimie.

[2] Zizza A., Sedile R., Bagordo F., Panico A., Guido M., Grassi T., Banchelli F., Grima P. (2023). Factors Associated with Pneumonia in Patients Hospitalized with COVID-19 and the Role of Vaccination. Vaccines.

[3] Haas E.J., Angulo F.J., McLaughlin J.M., Anis E., Singer S.R., Khan F., Brooks N., Smaja M., Mircus G., Pan K. (2021). Impact and effectiveness of mRNA BNT162b2 vaccine against SARS-CoV-2 infections and COVID-19 cases, hospitalisations, and deaths following a nationwide vaccination campaign in Israel: An observational study using national surveillance data. Lancet.

[4] Panico A., Lobreglio G., Bagordo F., Zizza A., De Donno A., Rosato C., Lazzari R., Chicone M., Indino F., Recchia V. (2022). Antibody Response in Healthcare Workers before and after the Third Dose of Anti-SARS-CoV-2 Vaccine: A Pilot Study. Vaccines.

[5] Grassi T., Lobreglio G., Panico A., Rosato C., Zizza A., Lazzari R., Chicone M., Indino F., Bagordo F. (2022). Kinetics of Humoral Immunity against SARS-CoV-2 in Healthcare Workers after the Third Dose of BNT162b2 mRNA Vaccine. Vaccines.

[6] World Health Organization (2020). WHO Laboratory Testing for 2019 Novel Coronavirus (2019-nCoV) in Suspected Human Cases. Interim Guidance. Geneva, Switzerland. https://www.who.int/publications/i/item/10665-331501.

[7] Binnicker M.J. (2020). Challenges and Controversies to Testing for COVID-19. J. Clin. Microbiol..

[8] Tom M.R., Mina M.J. (2020). To interpret the SARS-CoV-2 test, consider the cycle threshold value. Clin. Infect. Dis..

[9] Corman V.M., Landt O., Kaiser M., Molenkamp R., Meijer A., Chu D.K., Bleicker T., Brünink S., Schneider J., Schmidt M.L. (2020). Detection of 2019 novel coronavirus (2019-nCoV) by real-time RT-PCR. Eurosurveillance.

[10] Mishra B., Ranjan J., Purushotham P., Saha S., Payal P., Kar P., Das S., Deshmukh V. (2022). High proportion of low cycle threshold value as an early indicator of COVID-19 surge. J. Med. Virol..

[11] La Scola B., Le Bideau M., Andreani J., Hoang V.T., Grimaldier C., Colson P., Gautret P., Raoult D. (2020). Viral RNA load as determined by cell culture as a management tool for discharge of SARS-CoV-2 patients from infectious disease wards. Eur. J. Clin. Microbiol. Infect. Dis..

[12] Rao S.N., Manissero D., Steele V.R., Pareja J. (2020). A narrative systematic review of the clinical utility of cycle threshold values in the context of COVID-19. Infect. Dis. Ther..

[13] Bullard J., Dust K., Funk D., Strong J.E., Alexander D., Garnett L., Boodman C., Bello A., Hedley A., Schiffman Z. (2020). Predicting infectious SARS-CoV-2 from diagnostic samples. Clin. Infect. Dis..

[14] Singanayagam A., Patel M., Charlett A., Lopez Bernal J., Saliba V., Ellis J., Ladhani S., Zambon M., Gopal R. (2020). Duration of infectiousness and correlation with RT-PCR cycle threshold values in cases of COVID-19, England, January to May 2020. Eurosurveillance.

[15] Yu X., Sun S., Shi Y., Wang H., Zhao R., Sheng J. (2020). SARS-CoV-2 viral load in sputum correlates with risk of COVID-19 progression. Crit. Care.

[16] Han M.S., Seong M.W., Kim N., Shin S., Cho S.I., Park H., Kim T.S., Park S.S., Choi E.H. (2020). Viral RNA load in mildly symptomatic and asymptomatic children with COVID-19, Seoul, South Korea. Emerg. Infect. Dis..

[17] Young B.E., Ong S.W.X., Kalimuddin S., Low J.G., Tan S.Y., Loh J., Ng O.-T., Marimuthu K., Ang L.W., Mak T.M. (2020). Epidemiologic Features and Clinical Course of Patients Infected with SARS-CoV-2 in Singapore. J. Am. Med. Assoc..

[18] Wang Y., He Y., Tong J., Qin Y., Xie T., Li J., Li J., Xiang J., Cui Y., Higgs E.S. (2020). Characterization of an Asymptomatic Cohort of Severe Acute Respiratory Syndrome Coronavirus 2 (SARS-CoV-2) Infected Individuals Outside of Wuhan, China. Clin. Infect. Dis..

[19] Al Bayat S., Mundodan J., Hasnain S., Sallam M., Khogali H., Ali D., Alateeg S., Osama M., Elberdiny A., Al-Romaihi H. (2021). Can the cycle threshold (Ct) value of RT-PCR test for SARS CoV2 predict infectivity among close contacts?. J. Infect. Public Health.

[20] Cevik M., Tate M., Lloyd O., Maraolo A.E., Schafers J., Ho A. (2021). SARS-CoV-2, SARS-CoV, and MERS-CoV viral load dynamics, duration of viral shedding, and infectiousness: A systematic review and meta-analysis. Lancet Microbe.

[21] Skok K., Stelzl E., Trauner M., Kessler H.H., Lax S.F. (2021). Post-mortem viral dynamics and tropism in COVID-19 patients in correlation with organ damage. Virchows Arch..

[22] Abdulrahman A., Mallah S.I., Alawadhi A., Perna S., Janahi E.M., AlQahtani M.M. (2021). Association between RT-PCR Ct values and COVID-19 new daily cases: A multicenter cross-sectional study. Infez. Med..

[23] Platten M., Hoffmann D., Grosser R., Wisplinghoff F., Wisplinghoff H., Wiesmüller G., Schildgen O., Schildgen V. (2021). SARS-CoV-2, CT-Values, and Infectivity-Conclusions to Be Drawn from Side Observations. Viruses.

[24] Zou L., Ruan F., Huang M., Liang L., Huang H., Hong Z., Yu J., Kang M., Song Y., Xia J. (2020). SARS-CoV-2 viral load in upper respiratory specimens of infected patients. N. Engl. J. Med..

[25] Wölfel R., Corman V.M., Guggemos W., Seilmaier M., Zange S., Muller M.A., Niemeyer D., Jones T.C., Vollmar P., Rothe C. (2020). Virological assessment of hospitalized patients with COVID-2019. Nature.

[26] He X., Lau E.H.Y., Wu P., Deng X., Wang J., Hao X., Lau Y.C., Wong J.Y., Guan Y., Tan X. (2020). Temporal dynamics in viral shedding and transmissibility of COVID-19. Nat. Med..

[27] Wang Y., Tian H., Zhang L., Zhang M., Guo D., Wu W., Zhang X., Kan G.L., Jia L., Huo D. (2020). Reduction of secondary transmission of SARS-CoV-2 in households by face mask use, disinfection and social distancing: A cohort study in Beijing, China. BMJ Glob. Health.

[28] Cheng H.Y., Jian S.W., Liu D.P., Ng T.C., Huang W.T., Lin H.H. (2020). Contact tracing assessment of COVID-19 transmission dynamics in Taiwan and risk at different exposure periods before and after symptom onset. JAMA Intern. Med..

[29] Pan X., Chen D., Xia Y., Wu X., Li T., Ou X., Zhou L., Liu J. (2020). Asymptomatic cases in a family cluster with SARS-CoV-2 infection. Lancet Infect. Dis..

[30] To K.K.W., Tsang O.T.Y., Leung W.S., Tam A.R., Wu T.C., Lung D.C., Yip C.C.-Y., Cai J.-P., Chan J.M.-C., Chik T.S.-H. (2020). Temporal profiles of viral load in posterior oropharyngeal saliva samples and serum antibody responses during infection by SARS-CoV-2: An observational cohort study. Lancet Infect. Dis..

[31] Zhou F., Yu T., Du R., Fan G., Liu Y., Liu Z., Xiang J., Wang Y., Song B., Gu X. (2020). Clinical course and risk factors for mortality of adult inpatients with COVID-19 in Wuhan, China: A retrospective cohort study. Lancet.

[32] Liu W.D., Chang S.Y., Wang J.T., Tsai M.J., Hung C.C., Hsu C.L., Chang S.-C. (2020). Prolonged virus shedding even after seroconversion in a patient with COVID-19. J. Infect..

[33] Xiao A.T., Tong Y.X., Zhang S. (2020). Profile of RT-PCR for SARS-CoV-2: A preliminary study from 56 COVID-19 patients. Clin. Infect. Dis..

[34] Chen J., Qi T., Liu L., Ling Y., Qian Z., Li T., Li F., Xu Q., Zhang Y., Xu S. (2020). Clinical progression of patients with COVID-19 in Shanghai, China. J. Infect..

[35] Fang Z., Zhang Y., Hang C., Ai J., Li S., Zhang W. (2020). Comparisons of viral shedding time of SARS-CoV-2 of different samples in ICU and non-ICU patients. J. Infect..

[36] Seah I.Y.J., Anderson D.E., Kang A.E.Z., Wang L., Rao P., Young B., Lye D.C., Agrawal R. (2020). Assessing viral shedding and infectivity of tears in coronavirus disease 2019 (COVID-19) patients. Ophthalmology.

[37] Zhu L., Gong N., Liu B., Lu X., Chen D., Chen S., Shu H., Ma K., Xu X., Guo Z. (2020). Coronavirus disease 2019 pneumonia in immunosuppressed renal transplant recipients: A summary of 10 confirmed cases in Wuhan, China. Eur. Urol..

[38] Jefferson T., Spencer E.A., Brassey J., Heneghan C. (2021). Viral Cultures for Coronavirus Disease 2019 Infectivity Assessment: A Systematic Review. Clin Infect Dis.

[39] College of American Pathologists (2020). Laboratory General Checklist. GEN.54750, Nonwaived Testing Personnel Qualifications.

[40] Miller J.M., Binnicker M.J., Campbell S., Carroll K.C., Chapin K.C., Gilligan P.H., Gonzalez M.D., Jerris R.C., Kehl S.C., Patel R. (2018). A guide to utilization of the microbiology laboratory for diagnosis of infectious diseases: 2018 update by the Infectious Diseases Society of America and the American Society for Microbiology. Clin. Infect. Dis..

